# MUGA image artefacts caused by metallic injection ports in breast reconstruction tissue expanders: a report of two breast cancer patients

**DOI:** 10.1259/bjrcr.20150421

**Published:** 2016-07-28

**Authors:** William Makis

**Affiliations:** Department of Diagnostic Imaging, Cross Cancer Institute, Edmonton, Canada

## Abstract

Expander-based breast reconstruction is a popular form of post-mastectomy reconstruction and involves the temporary subcutaneous implantation of breast tissue expanders that require periodic, incremental inflation with sterile saline by injection until the desired amount of tissue is developed. One type of tissue expander injection port system currently on the market is made of titanium and rare-earth magnets that enhance injection accuracy. These highly dense metallic materials, however, can cause attenuation artefacts on multiple gated acquisition cardiac studies. In this report, we present the cases of two breast cancer patients with artefacts on multiple gated acquisition scans, characteristic of these tissue breast expanders.

## Case Report

### Patient 1

**Figure 1.**
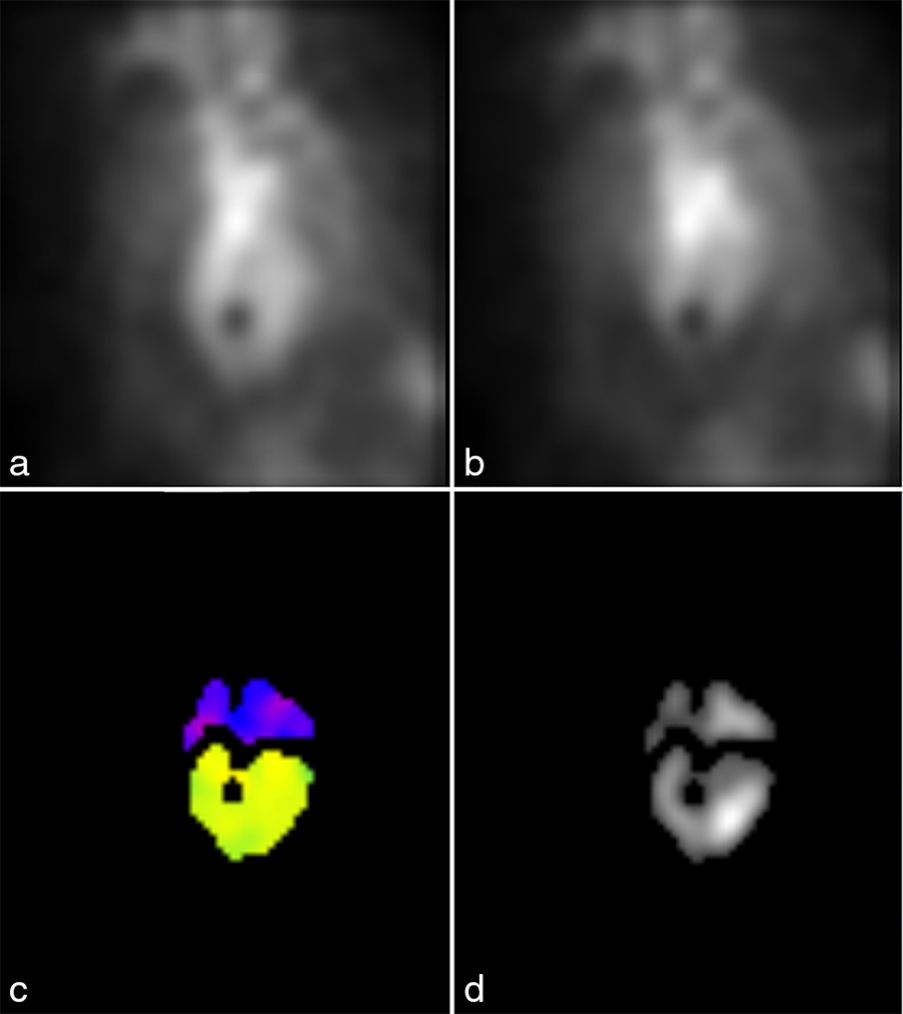
Patient 1. The left anterior oblique view images at end diastole (a) and end systole (b) show a small round photopenic defect overlying the mid-septum. A round focus of absent counts in the region of the septum, measuring approximately 1.5 cm, was also identified on the phase (c) and amplitude (d) parametric images.

**Figure 2.**
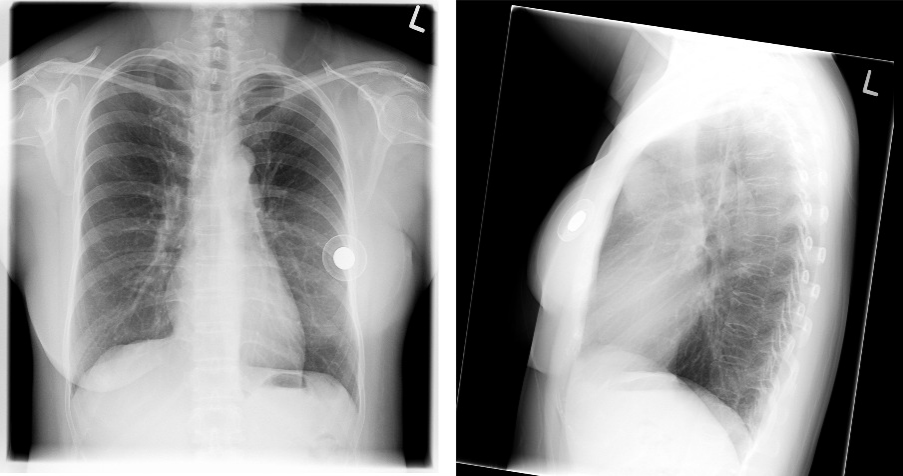
Patient 1. A chest radiograph revealed a dense ring-like object in the region of the left breast, which was the metallic injection port of the left breast tissue expander.

A 49-year-old female was diagnosed with a pT2N0 (Stage IIA) 2.6 cm invasive ductal carcinoma of the left breast (ER+, PR+, HER-2+, Grade 3/3). She had left mastectomy and 1 month later was booked for a multiple gated acquisition (MUGA) scan to assess her baseline cardiac function before starting adjuvant chemotherapy with trastuzumab, carboplatin and docetaxel. The left anterior oblique (LAO) view images (Figure 1a,b) revealed a small round photopenic defect overlying the septum, which did not change location on the dynamic images. A round focus of absent counts in the region of the septum, measuring approximately 1.5 cm, was also identified on the phase and amplitude parametric images (Figure 1c,d). The ejection fraction was calculated at 66% and was in the normal range. A chest radiograph revealed a dense ring-like object in the region of the left breast (Figure 2). 1 month earlier, the patient had a skin-sparing left mastectomy with immediate reconstruction. This was achieved by the placement of a breast tissue expander that was inserted beneath the left pectoralis major muscle (Allergan style 133SV-14-T anatomic saline tissue expander of 375 ml nominal volume). The dense ring-like object seen on chest X-ray was a MAGNA-SITE® (Allergan, Santa Barbara, CA) integrated injection port in the tissue expander, which contains a puncture-proof titanium needle guard and a rare-earth permanent magnet and which is used in conjunction with the MAGNA-FINDER® external locating device (which also contains a rare-earth permanent magnet) for an accurate injection system. The cause of the photopenic artefacts on the MUGA study was the metallic injection port of the left breast tissue expander.

### Patient 2

**Figure 3.**
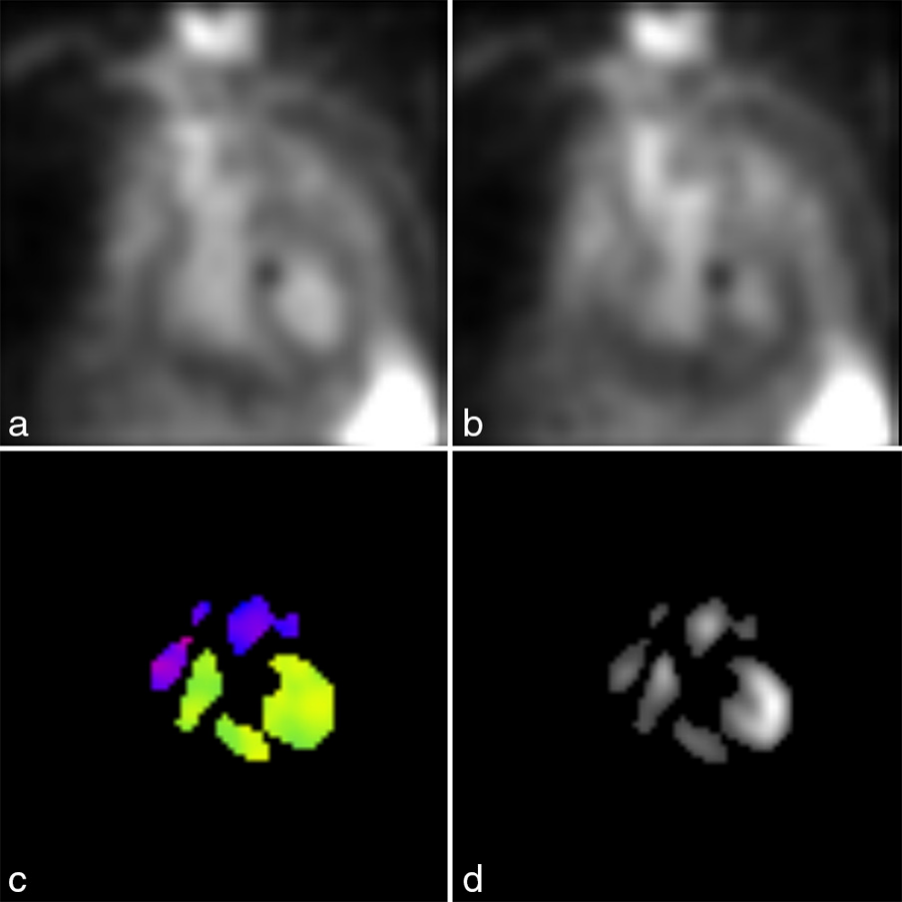
Patient 2. The left anterior oblique view images at end diastole (a) and end systole (b) show a small round photopenic defect overlying the upper part of the septum. There was also a semicircular focus of absent counts in the upper part of the septum extending into the left ventricle on the phase (c) and amplitude (d) parametric images.

**Figure 4.**
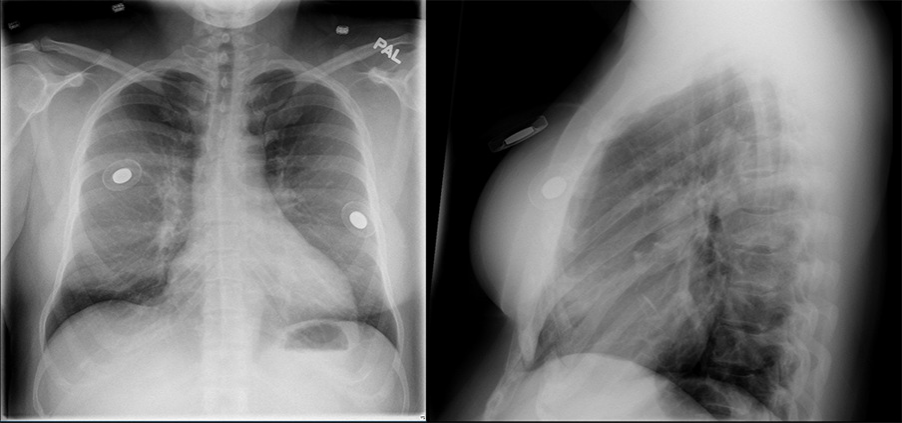
Patient 2. A chest radiograph revealed two dense ring-like objects in the regions of the breasts, which were the metallic injection ports of the breast tissue expanders.

**Figure 5.**
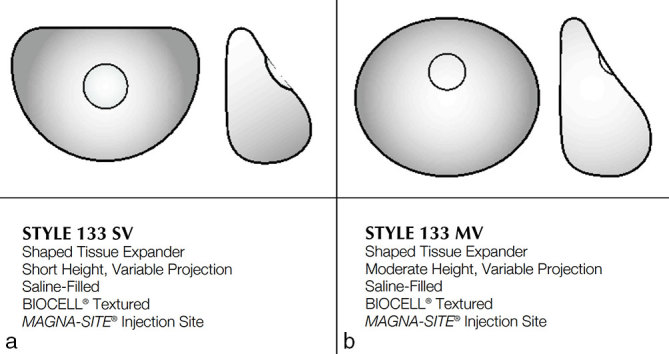
A schematic of the patients’ breast reconstruction tissue expanders, showing the shape of the tissue expander and the central location of the dense metal integrated injection port. (a) Patient 1, (b) patient 2. Reproduced from Allergan^1^ with permission from Allergan Inc.

**Figure 6.**
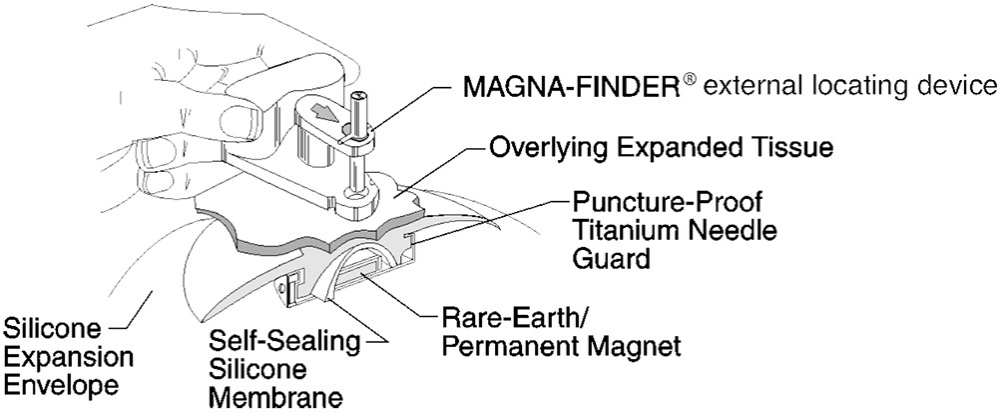
A schematic of the MAGNA-SITE® integrated injection port in the tissue expander, which contains a puncture proof titanium needle guard and a rare-earth permanent magnet, which is used in conjunction with the MAGNA-FINDER® external locating device (that also contains a rare-earth permanent magnet) for an accurate injection system. Reproduced from Allergan^1^ with permission from Allergan Inc.

A 41-year-old female was diagnosed with a 0.7 cm invasive ductal carcinoma of the left breast (ER+, PR–, HER2+, Grade 3/3) as well as a right breast 5.5 cm ductal carcinoma *in situ* (Grade 3/3). She had a skin-sparing bilateral mastectomy with insertion of breast tissue expanders (Allergan style 133MV-14-T 500 ml with an initial fill volume of 250 ml for each side). These expanders also have a MAGNA-SITE integrated injection port in the tissue expander. MUGA scans performed at 2, 5 and 8 months after expander insertion showed a photopenic defect overlying the upper part of the septum (Figure 3a,b). There was also a semicircular focus of absent counts in the upper part of the septum extending into the left ventricle on the phase and amplitude parametric images (Figure 3c,d). A chest radiograph revealed two dense ring-like objects in the breast regions (Figure 4), which were the tissue expander metallic injection ports. The tissue expander design for the two patients is shown in Figure 5 and the metallic injection port in Figure 6.

## Discussion

Expander-based breast reconstruction is a popular form of post-mastectomy reconstruction and comprises over 60% of all breast reconstruction procedures according to the American Society of Plastic Surgeons.^2^ Tissue expanders are intended for temporary subcutaneous implantation and require periodic, incremental inflation with sterile saline for injection until the desired amount of tissue is developed. The breast tissue expanders of both these patients had an injection port that contained a titanium needle guard, as well as a rare-earth magnet for accurate injection of saline.^3,4^ The dense metallic injection port caused attenuation of the γ photons during the MUGA acquisition of the LAO view, resulting in fixed photopenic defects on all of the dynamic images, as well as circular or semicircular foci of absent counts on the phase and amplitude parametric images. Small focal attenuation artefacts on planar MUGA studies can be caused by any metallic object overlying the heart, including necklaces, pendants or nipple piercings. Other pitfalls of MUGA imaging include large circular attenuation defects that can be caused by breast prostheses,^5^ large breasts, pericardial effusion, pericardial cysts or tumours, epicardial fat, pleural fluid, mediastinal adenopathy or tumours, and mediastinal fat;^6–11^ chamber filling defects, which include atrial myoma, cardiac tumours (including metastases), prominent papillary muscle or trabeculae and thrombi; saccular deformities of the left ventricle such as aneurysm, diverticulum, ectopic spleen and localized herniation through a partial pericardial defect; true aneurysms as well as false aneurysms.^11–15^ To our knowledge, MUGA artefacts in patients with breast cancer caused by breast tissue expanders with metallic injection ports have not been previously described.

## Learning Points

1. Expander-based breast reconstruction is a popular form of post-mastectomy reconstruction.
2. Tissue breast expanders have dense metallic injection ports to enhance injection accuracy.
3. These dense metallic injection ports cause attenuation artefacts on MUGA LAO dynamic images, as well as phase and amplitude parametric images.

## Consent

Informed consent to publish this case report (including images and data) was obtained and is held on record.
